# A case of Myhre syndrome mimicking juvenile scleroderma

**DOI:** 10.1186/s12969-020-00466-1

**Published:** 2020-09-11

**Authors:** Barbara Jensen, Rebecca James, Ying Hong, Ebun Omoyinmi, Clarissa Pilkington, Neil J. Sebire, Kevin J. Howell, Paul A. Brogan, Despina Eleftheriou

**Affiliations:** 1 Infection, Immunity and Inflammation Research and Teaching Department, UCL Great Ormond Street Institute of Child Health, 30 Guilford Street, London, WC1N 1EH UK; 2 Paediatric Rheumatology Department, Queensland Children’s Hospital, Brisbane, Australia; 3 Paediatric Rheumatology Department, Great Ormond Street Hospital for Children NHS Foundation Trust, London, UK; 4 Histopathology Department, Great Ormond Street Hospital for Children NHS Foundation Trust, London, UK; 5 Microvascular Diagnostics, UCL Institute of Immunity and Transplantation, Royal Free Hospital, London, UK; 6 Centre for Adolescent Rheumatology Versus Arthritis at UCL, London, UK

**Keywords:** Scleroderma, Myhre syndrome, *SMAD4*

## Abstract

**Background:**

Myhre syndrome is a genetic disorder caused by gain of function mutations in the SMAD Family Member 4 *(SMAD4)* gene, resulting in progressive, proliferative skin and organ fibrosis. Skin thickening and joint contractures are often the main presenting features of the disease and may be mistaken for juvenile scleroderma.

**Case presentation:**

We report a case of a 13 year-old female presenting with widespread skin thickening and joint contractures from infancy. She was diagnosed with diffuse cutaneous systemic sclerosis, and treatment with corticosteroids and subcutaneous methotrexate recommended. There was however disease progression prompting genetic testing. This identified a rare heterozygous pathogenic variant c.1499 T > C (p.Ile500Thr) in the *SMAD4* gene, suggesting a diagnosis of Myhre syndrome. Securing a molecular diagnosis in this case allowed the cessation of immunosuppression, thus reducing the burden of unnecessary and potentially harmful treatment, and allowing genetic counselling.

**Conclusion:**

Myhre Syndrome is a rare genetic mimic of scleroderma that should be considered alongside several other monogenic diseases presenting with pathological fibrosis from early in life. We highlight this case to provide an overview of these genetic mimics of scleroderma, and highlight the molecular pathways that can lead to pathological fibrosis. This may provide clues to the pathogenesis of sporadic juvenile scleroderma, and could suggest novel therapeutic targets.

## Background

Myhre syndrome is a genetic disorder often presenting in infancy, caused by a gain of function mutation in the SMAD family member 4 (*SMAD4*) gene causing progressive, proliferative fibrosis, occurring spontaneously or following trauma, in addition to a unique set of clinical phenotypic features described below [1–4]. Clinical manifestations of Myhre syndrome include: cardiovascular involvement in up to 70% of patients (congenital heart defects, long- and short-segment stenosis of the aorta and peripheral arteries, pericardial effusion, constrictive pericarditis, restrictive cardiomyopathy, and arterial hypertension); respiratory manifestations (choanal stenosis, laryngotracheal narrowing, obstructive airway disease, or restrictive pulmonary disease); gastrointestinal symptoms (pyloric stenosis, duodenal strictures, severe constipation); hearing loss, mild to moderate development delay, dysmorphic features and skin involvement (skin sclerosis, particularly involving the hands and extensor surfaces) leading to joint contractures [1, 5–12]. Patients presenting with predominantly skin sclerosis and contractures, cardiovascular involvement may be misdiagnosed as a having systemic sclerosis (SSc) despite the presence of other atypical features for SSc such as hearing loss and developmental delay thus causing unnecessary exposure to immunosuppression. Herein, we present a case of a 13 year-old female considered as having diffuse cutaneous systemic sclerosis, who was subsequently identified to have Myhre syndrome caused by a previously well described heterozygous c.1499 T > C variant in *SMAD4*. We discuss the therapeutic implications of establishing a genetic diagnosis in this case and provide an overview of genetic mimics of scleroderma.

## Case presentation

**Fig. 1:**
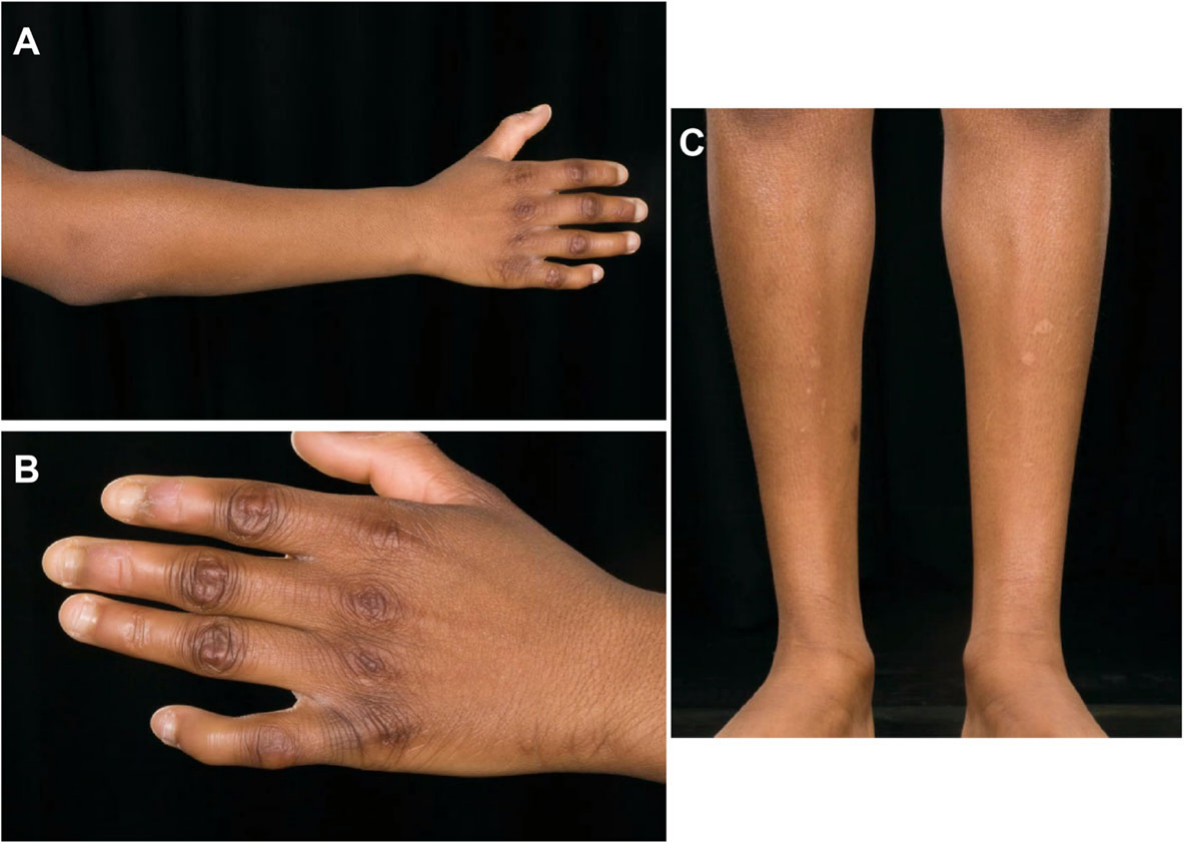
Cutaneous and skeletal manifestations of the 13 year old patient with Myhre syndrome we describe in this report. **a-b** Multiple joint contractures and clinodactyly. **c** Extensive skin thickening and muscle wasting of lower limbs in same patient

A 13 year-old girl of Black African decent was referred to the scleroderma services of the rheumatology department at Great Ormond Street Hospital for Children NHS Foundation Trust, London for a second opinion with history of extensive skin thickening and widespread joint contractures, which started in infancy at the age of 9 months (Fig. 1). The skin changes started in her lower limps and over the course of 2 years spread to the arms and trunk. The joint contractures were noted approximately 2 years after the initial skin changes were observed. There was history suggestive of mild Raynaud’s phenomenon, but no digital ulceration, gastrointestinal, or respiratory symptoms of note. She was born at term with no neonatal complications. She had a past medical history of: valvar and supravalvar pulmonary artery stenosis requiring serial balloon dilatation; mild developmental delay; and conductive hearing loss. Microarray-based comparative genomic hybridization was used to exclude chromosomal abnormalities that could explain her presentation and was normal. There was no history of cancer in the immediate family.

**Fig. 2:**
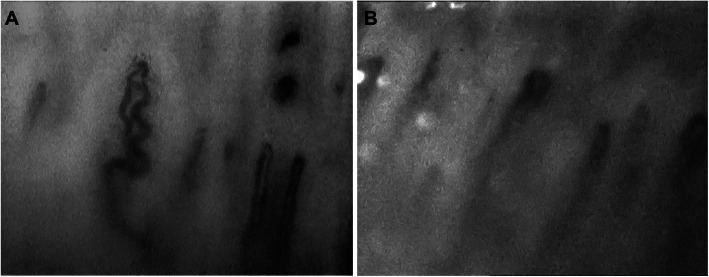
Nailfold capillaroscopy of the 13 year old patient with Myhre syndrome we describe in this report. **a-b** Abnormal nailfold capillary patterns with small microbleeds, very tortuous loops and mild dilatation in a patient with Myhre syndrome suggesting an evolving microangiopathy

**Fig. 3:**
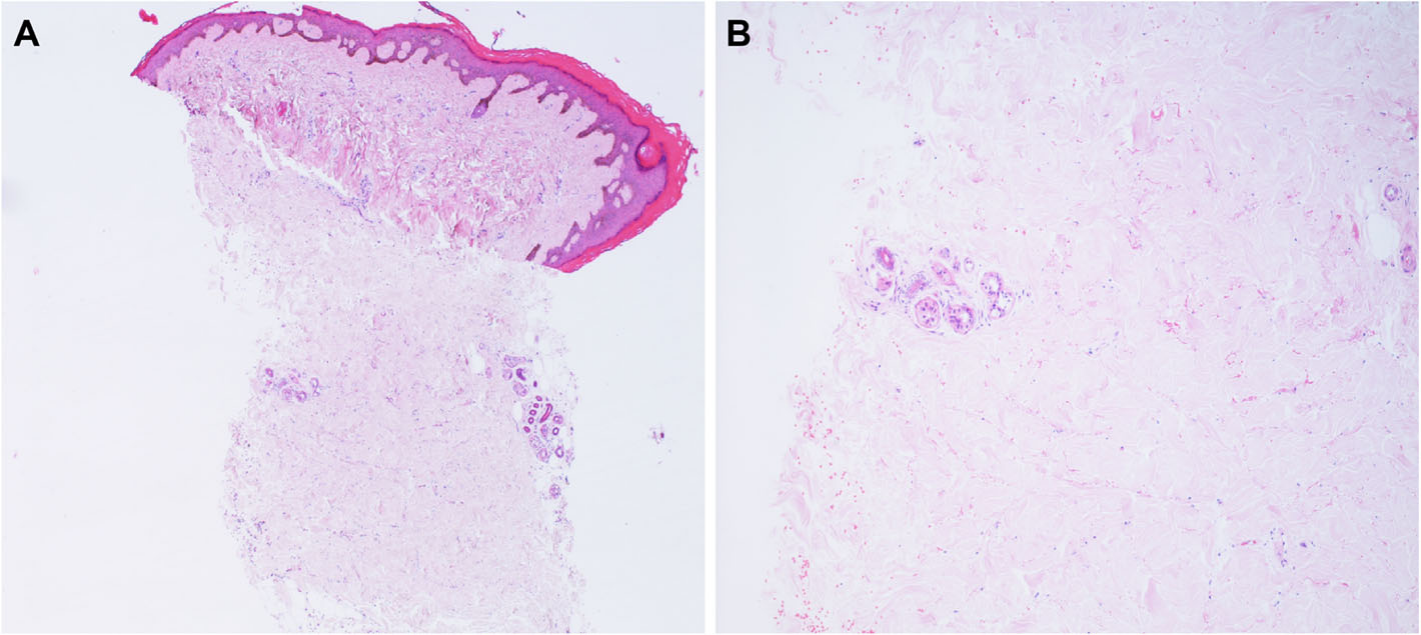
Skin histology of the 13 year old patient with Myhre syndrome mimicking juvenile scleroderma we describe in this report. **a-b** Photomicrographs of skin punch biopsy containing epidermis, dermis and superficial subcutis. There is no significant lichenoid reaction or inflammatory infiltrate (L) but the dermis shows marked replacement by hypocellular, hyalinised areas of bland collagen(R). There are no other specific features and the adnexal structures remain in this biopsy. (H&E, original magnifications Lx40 and Rx100)

Clinical examination revealed diffusely thickened skin affecting the full length of her limbs and trunk, but sparing her face; weight 41 kg (25th centile for age), height 139 cm (2nd centile for age). She was normotensive at time of review. Delayed puberty was noted and the patient had no menarche at the age of 13 years old. There were multiple joint contractures, but no active arthritis. Cutaneous telangiectasia, fingertip ulceration and calcinosis were absent. She was also noted to have mild dysmorphic features: small eyes and ears, a broad nasal tip, a long and prominent chin, bilateral clinodactyly and mild two-three toe syndactyly. Nailfold capillaroscopy was abnormal, with evidence of dilated capillary loops, tortuosity, micro-bleeding, and widespread dropout in a pattern compatible with scleroderma-spectrum connective tissue disease **(**Fig. 2). Digital thermography demonstrated cold baseline cutaneous temperature of the peripheries, with some of the fingers remaining cool long after cold challenge. She tested weakly positive for antinuclear antibodies (ANA at 1:160, homogenous pattern); negative for dsDNA antibodies, rheumatoid factor and extranuclear antibodies. Complement function (alternate and classical pathways) was normal, as were levels of C3, C4, and C1q. Erythrocyte sedimentation rate and C-reactive protein were repeatedly within normal limits. Echocardiography revealed mild persistent pulmonary stenosis, a small left pulmonary artery, mild coarctation of the aorta, mild biventricular hypertrophy, but no evidence of pulmonary hypertension. Barium swallow was normal. A skeletal survey revealed advanced bone age, but no evidence of skeletal dysplasia. A skin biopsy was performed, with histology revealing hyperkeratotic epidermis, and fibrotic dermis with areas of hyalinization; adnexal structures were sparse with absence of pilosebaceous units **(**Fig. 3**).** These histological features are typically encountered in scleroderma histopathology with the exception of the hyperkeratotic epidermis, which is less often seen [11, 13, 14]. She was diagnosed with diffuse cutaneous systemic sclerosis, and treatment with oral prednisolone 2 mg/kg/day for 6 weeks, and subcutaneous methotrexate (15 mg/m^2^/week) started. There was deterioration in joint contractures (further loss of range of movement) and spreading of skin changes observed despite treatment. When reviewed for a second opinion at GOSH, a genetic diagnosis was suspected and genetic testing via Sanger sequencing was undertaken for some conditions that cause skin thickening, dysmorphic features and congenital heart disease. Genetic testing revealed a previously well described rare heterozygous c.1499 T > C (p.lle500Thr) class 5 variant in SMAD4 [12], suggesting a diagnosis of Myhre syndrome. Testing for variants in other relevant genes pertinent to phenotype (including *PTPN11, LMNA, and MMP14*) revealed no other pathogenic variants [15–26]. Parental testing confirmed this variant arose de novo in the proband. All immunosuppression was subsequently stopped, genetic counselling was provided, and the prognosis of Myhre syndrome was discussed with the patient and family.

## Discussion

We present the case of a 13 year-old with a scleroderma-like condition, ultimately diagnosed with Myhre syndrome, a genetic disorder that may mimic juvenile scleroderma (Supplemental Table 1). Securing a molecular diagnosis in this case allowed the cessation of immunosuppression thus reducing the burden of unnecessary toxic exposure to glucocorticoids, and other ineffective immunosuppressive treatments; and facilitated genetic counselling, and prognostication. This also had implications for long term follow up as patients with Myhre syndrome require close surveillance for detection of any malignancy in view of increased risk of cancer reported in these patients [5, 12, 27]. We therefore highlight this case to raise awareness of a growing number of monogenic fibrotic disorders mimicking juvenile scleroderma which need to be considered in patients with cutaneous fibrosis beginning early in life (Table 1).

**Table 1:** Monogenic disorders with a scleroderma-like phenotype. The clinical features have been summarised as described by the Online Mendelian Inheritance in Man (OMIM) [28] and Genetics Home Reference databases [29]

Disease	Inheritance	Gene	Clinical Features
Hutchinson-Gilford Progeria	AD, AR	*LMNA*	**Skin:** Sclerodermatous skin disease, loss of subcutaneous fat (lipodystrophy) **Skeletal:** Osteoporosis, joint restrictions, joint abnormalities **Cardiovascular:** Atherosclerosis **Other:** Prematurely aged appearance, postnatal onset growth retardation, hair loss (alopecia)
Werner syndrome	AR	*WRN*	**Skin:** Sclerodermatous skin disease, subcutaneous calcification, ulceration **Skeletal:** Osteoporosis **Cardiovascular:** Premature arteriosclerosis **Endocrine:** Diabetes mellitus, hypogonadism **Other:** Prematurely aged appearance, short stature, alopecia, juvenile cataracts
Rothmund Thomson syndrome	AR	*RECQL4*	**Skin:** Erythematous thickened skin lesions in infancy, poikiloderma (atrophic plaques with telangiectasia), telangiectasia, atrophy, sun sensitivity **Skeletal:** Osteoporosis **Central Nervous System:** Mental retardation (rare) **Endocrine:** Hypogonadism **Other:** Prematurely aged appearance, short stature, alopecia, premature greying of hair, increased risk of malignant disease
Mandibular hypoplasia, deafness, progeroid features and lipodystrophy syndrome	AD	*POLD1*	**Skin:** Sclerodermatous skin disease, telangiectasias, atrophy, lipodystrophy **Skeletal:** Osteoporosis, joint contractures **Endocrine:** Insulin resistance, diabetes mellitus **Other:** Prematurely aged appearance, mandibular hypoplasia, sensorineural deafness, hepatomegaly, hepatic steatosis
Nestor-Guillermo Progeria Syndrome	AR	*BANF1*	**Skin:** Sclerodermatous skin disease (patchy) and hyperpigmentation **Skeletal:** Joint stiffness, joint contractures, osteoporosis, osteolysis **Cardiovascular:** Sinus tachycardia, prominent subcutaneous venous patterning, pulmonary hypertension **Other:** Prematurely aged appearance, short stature, lipoatrophy
Keppen-Lubinsky syndrome	AD	*KCNJ6*	**Skin:** Lipodystrophy, wrinkled appearance **Skeletal:** Joint contractures **Central Nervous System:** Severe mental retardation, delayed psychomotor development, hypertonia, hyperreflexia **Other:** Prematurely aged appearance, generalised lipodystrophy
Fontaine Progeroid Syndrome	AD	*SLC25A24*	**Skin:** Wrinkled skin, lipodystrophy, sclerodermatous skin disease **Skeletal:** Low bone density, delayed bone age **Cardiovascular:** Pulmonary artery hypertension, aortic ectasia **Other:** Prematurely aged appearance, short stature, intrauterine growth retardation
Cockayne Syndrome, Type A	AR	*ERCC8*	**Skin:** Cutaneous photosensitivity, scarred, pigmented, atrophy, reduced subcutaneous adipose tissue, sclerodermatous skin disease **Skeletal:** Flexion contractures, mild-to-moderate joint limitations **Cardiovascular:** Hypertension **Neurological:** Impaired or delayed neural development, mental retardation **Other:** Prematurely aged appearance, cachectic dwarfism, intrauterine growth retardation, sensorineural hearing loss, vision complications, tooth decay, hepatomegaly, splenomegaly, decreased subcutaneous adipose tissue
Ataxia-telangiectasia	AR	*ATM*	**Skin:** Sclerodermatous skin disease, progeric skin changes, cutaneous telangiectasia, cafe-au-lait spots **Respiratory:** Bronchitis, bronchiectasis **Neurological:** Cerebellar ataxia, cerebellar cortical degeneration, oculomotor abnormalities, seizures, choreoathetosis, dystonia, reduced/absent deep tendon reflexes **Other:** Short stature
Myhre syndrome	AD	*SMAD4*	**Skin:** Sclerodermatous skin disease **Skeletal:** Skeletal abnormalities, joint restrictions **Cardiovascular:** Hypertension, congenital heart defects, aortic stenosis, aortic coarctation, pericardial fibrosis **Respiratory:** Laryngotracheal stenosis, respiratory failure **Neurological:** Mental retardation, delayed language and motor skill development, behavioural issues (autistic-like) **Other:** Dysmorphic facial features, short stature, hearing loss, generalised muscle hypertrophy
Stiff skin syndrome	AD	*FBN1*	**Skin:** Sclerodermatous skin disease (diffuse), lipodystrophy **Skeletal:** Joint restrictions, flexion contractures **Other:** Muscle weakness
Pigmentary hypertrichosis and non-autoimmune insulin-dependent diabetes mellitus (PHID)	AR	*SLC29A3*	**Skin:** Hyperpigmented and hypertrichotic skin lesions on lower body, sclerodermatous skin disease **Skeletal:** Joint contractures (elbows, fingers and toes) **Abdomen:** Hepatomegaly, diabetes mellitus (insulin-dependent), splenomegaly **Other:** Short stature, hearing loss
Reynolds syndrome	AD	*LBR*	**Skin:** Sclerodermatous skin disease (tightened and shiny skin over the forearms and hands), sclerodactyly, calcinosis cutis, generalized darkening **Other:** Raynaud phenomenon, hepatomegaly, primary biliary cirrhosis, splenomegaly, esophageal dysfunction
Chronic atypical neutrophilic dermatosis with lipodystrophy and elevated temperature (CANDLE) syndrome /Nakajo-Nishimura Syndrome	AR	*PSMB8*	**Skin:** Erythematous nodular skin lesions and plaques on the face and extremities, dry, stiff, lipodystrophy **Skeletal:** Joint contractures (elbow, fingers/hands, toes, feet), joint pain **Muscle:** Lipodystrophy, muscle weakness **Other:** Poor growth, hepatomegaly, splenomegaly
Mucolipidosis III gamma	AR	*GNPTG*	**Skin:** Sclerodermatous skin disease **Skeletal:** Joint restrictions, joint stiffness, joint pain **Cardiovascular:** Aortic valve thickening, aortic stenosis **Neurological:** Mental retardation **Other:** Short stature
Hurler-Scheie syndrome / Mucopolysaccharidosis Ih/s	AR	*IDUA*	**Skin:** Sclerodermatous skin disease **Skeletal:** Joint stiffness, dysostosis multiplex **Cardiovascular:** Thickened mitral valve leaflets, aortic valve thickening, dilated left atrium, dilated left ventricle, mild pulmonary hypertension **Respiratory:** Frequent respiratory infections, nasopharyngeal obstruction, tracheal stenosis **Abdomen:** Umbilical hernia, hepatomegaly, splenomegaly **Neurological:** Pachymeningitis cervicalis **Other:** Short stature, corneal clouding
Zimmermann-Laband Syndrome 1	AD	*KCNH1*	**Skin:** Dry, sclerodermatous skin disease **Skeletal:** Scoliosis, hypoplastic distal phalanges (hands and feet), hyperextensible joints **Abdomen:** Hepatosplenomegaly, splenomegaly, umbilical hernia **Cardiovascular:** Cardiomyopathy, patent ductus arteriosus, aortic root dilatation, aortic arch dilatation **Muscle:** Poor muscle bulk **Neurological:** Hypotonia, seizures, mental retardation **Other:** Gingival fibromatosis, dysplastic or absent nails, hirsutism, abnormalities of the cartilage of the nose and/or ears
Buschke-Ollendorff syndrome	AD	*LEMD3*	**Skin:** Subcutaneous nontender firm nodules, subcutaneous connective tissue nevi, elastin-rich connective tissue nevi (elastoma), collagen-rich connective tissue nevi (dermatofibrosis lenticularis disseminata) **Skeletal:** Osteopoikilosis, joint stiffness, osteosclerosis, melorheostosis
Growth Retardation, Alopecia, Pseudoanodontia and Optic Atrophy (GAPO) Syndrome	AR	*ANTXR1*	**Skin:** Sclerodermatous skin disease, redundant, prominent scalp veins, epidermal inclusion cyst **Skeletal:** Delayed bone age **Other:** Growth retardation, alopecia, pseudoanodontia, umbilical hernia, hepatomegaly
Crouzon Syndrome with acanthosis nigricans	AD	*FGFR3*	**Skin:** Hyperpigmentation, acanthosis nigricans, melanocytic nevi, hypertrophy, sclerodermatous skin disease, redundant skin folds **Skeletal:** Craniosynostosis
Frontometaphyseal dysplasia 2	AD	*MAP 3 K7*	**Skin:** Keloid formation, sclerodermatous skin disease **Skeletal:** Skeletal abnormalities, joint contractures **Cardiovascular:** Patent ductus arteriosus, bicuspid aortic valve, aortic root dilation, pulmonary valve stenosis **Respiratory:** Congenital stridor, subglottic stenosis, tracheal stenosis
Premature aging syndrome, Penttinen type	AD	*PDGFRB*	**Skin:** Progressive cutaneous atrophy, thin translucent skin with prominent venous patterning, hypertrophic keloid-like lesions, skin retraction, sclerodermatous skin disease, lipoatrophy **Skeletal:** Delayed bone maturation, osteopenia, joint contractures
Farber Lipogranulomatosis	AR	*ASAH1*	**Skin:** Early-onset subcutaneous nodules, lipogranulomatosis **Skeletal:** Painful and progressively deformed joints, arthritis **Respiratory:** Laryngeal nodules **Abdomen:** Hepatomegaly, splenomegaly **Neurological:** Irritability, motor retardation, mental retardation **Other:** Hoarseness by laryngeal involvement
Amyloidosis, Primary Localised cutaneous, 3 (PLCA3)	AR	*GPNMB*	**Skin:** Amyloid disposition in the skin, hyper- and hypo-pigmented macules, mild pruritis, dry skin
Carney Complex, Type 1	AD	*PRKAR1A*	**Skin:** Cutaneous tumors, profuse pigmented skin lesions, nevi **Cardiovascular:** Tumors (atrial), ventricular myxoma, congestive heart failure **Endocrine:** Tumors, pigmented micronodular adrenal dysplasia, Cushing disease, acromegaly, thyroid follicular hyperplasia **Other:** Neoplasia, myxoid subcutaneous tumors, primary adrenocortical nodular hyperplasia, testicular Sertoli cell tumor (calcified), pituitary adenoma, mammary ductal fibroadenoma, schwannoma, psammomatous melanotic schwannomas, thyroid carcinoma, pheochromocytoma
Porphyria cutanea tarda, Porphyria, hepatoerythropoietic	AD, AR	*UROD*	**Skin:** Sclerodermatous skin disease (diffuse), increased mechanical skin fragility after sunlight exposure (photosensitivity), vesicles, bullae and blisters on exposed areas of skin, hyperpigmentation on sun-exposed skin **Abdomen:** Hepatic hemosiderosis, hepatic cirrhosis, liver biopsy shows red autofluorescence and needle-like cytoplasmic inclusion bodies **Other:** Neoplasia, increased incidence of hepatocellular carcinoma
Phenylketonuria, non-PKU mild Hyperphenylalaninemia	AR	*PAH*	**Skin:** Sclerodermatous skin disease, pale pigmentation, dry, eczema **Neurological:** Seizures, delayed development, mental retardation, behavioural problems and psychiatric disorders **Other:** Head, microcephaly, cataracts
Porphyria, congenital erythropoietic	AR	*UROS*	**Skin:** Sclerodermatous skin disease, photosensitivity, blistering and scarring, hyperpigmentation, hypopigmentation **Skeletal:** Osteolysis, osteopenia, finger contractures **Other:** Short stature, conjunctivitis, corneal scarring, hypertrichosis, alopecia, porphyrin-rich gallstones, splenomegaly
Multicentric osteolysis, nodulosis and arthropathy (MONA)	AR	*MMP2*	**Skin:** Subcutaneous nodules (interphalangeal joints, knees, feet, elbows, pretibial), hyperpigmented erythematous lesions **Skeletal:** Osteoporosis, flexion contractures
Winchester syndrome	AR	*MMP14*	**Skin:** Sclerodermatous skin disease (patchy, dark, leathery) **Skeletal:** Osteopenia, osteoporosis, arthropathy, joint restrictions **Cardiovascular:** Heart abnormalities **Other:** Corneal opacity, hypertrichosis, overgrowth of the gums, coarse facial features
Multisystemic fibrosis-like hereditary fibrosing poikiloderma with tendon contractures, myopathy and pulmonary fibrosis (POIKTMP)	AD	*FAM111B*	**Skin:** Congenital poikiloderma (face and exposed skin), telangiectatic lesions, eczema-like lesions, epidermal atrophy **Respiratory:** Interstitial pulmonary fibrosis **Muscle:** Tendon contractures, muscle weakness, myopathy **Other:** Congenital poikiloderma on face
Weill-Marchesani syndrome 1	AR	*ADAMTS10*	**Skin**: Sclerodermatous skin disease **Skeletal:** Joint stiffness, joint restrictions **Cardiovascular:** Heart defects, aortic valve stenosis, pulmonary valve stenosis, ductus arteriosus, ventricular septal defect **Neurological:** Mild mental retardation **Other:** Short stature, brachydactyly, eye anomalies
Weill-Marchesani syndrome 4 (WMS-like syndrome)	AR	*ADAMTS17*	**Skin:** Sclerodermatous skin disease **Skeletal:** Joint stiffness **Cardiovascular:** Cardiac defects (uncommon) **Other:** Short stature, severe myopia, acute and/or chronic glaucoma, cataract
Frank-Ter Haar Syndrome	AR	*SH3PXD2B*	**Skin:** Sclerodermatous skin disease (face), acne conglobata **Skeletal:** Osteolysis, osteopenia, osteoporosis, shortened bowed long bones, flexion deformities of fingers **Other:** Growth retardation, glaucoma, brachycephaly, wide fontanels, prominent forehead, hypertelorism, prominent eyes, malocclusion
Geleophysic dysplasia 3	AD	*LTBP3*	**Skin:** Sclerodermatous skin disease **Skeletal:** Joint restrictions, delayed bone age **Cardiovascular:** Pulmonary hypertension **Respiratory:** Dyspnea, tracheal stenosis, respiratory failure **Other:** Short stature, marked brachydactyly, hepatomegaly
Geleophysic dysplasia 1	AR	*ADAMTSL2*	**Skin:** Sclerodermatous skin disease **Skeletal:** Osteopenia, shortened long tubular bones, short hands and feet, joint contractures, joint restrictions, delayed bone age **Cardiovascular:** Progressive cardiac valvular thickening, cardiac failure, mitral stenosis, tricuspid stenosis, aortic stenosis **Respiratory:** Tracheal stenosis, respiratory insufficiency **Neurological:** Developmental delay, seizures **Other:** Short stature, ‘happy’ appearance with full cheeks, shortened nose, wide mouth, hepatomegaly
Mucolipidosis II Alpha/Beta	AR	*GNPTAB*	**Skin:** Sclerodermatous skin disease, cavernous hemangioma **Skeletal:** Skeletal abnormalities, moderate joint restrictions, osteopenia **Cardiovascular:** Cardiomegaly, congestive heart failure, hypertrophic cardiomyopathy, cardiac murmur, aortic insufficiency **Respiratory:** Recurrent bronchitis, recurrent pneumonia **Abdomen:** Umbilical hernia, hepatomegaly **Neurological:** Developmental delay, severe psychomotor retardation **Other:** Progressive failure to thrive, Hurler-like body configuration, marked growth retardation, coarse facial features, abdominal protuberance, hoarse voice
Hypertrophic Osteoarthropathy, Primary, Autosomal Recessive 1 / Cranioosteoarthropathy	AR	*HPGD*	**Skin:** Sclerodermatous skin disease, pachydermia, furrowed, oily, seborrhea, redundant, palmoplantar hyperkeratosis, eczema **Skeletal:** Digital clubbing, osteoarthropathy, arthralgia, arthritis, swollen joints, decreased joint mobility, osteopenia, osteoporosis **Cardiovascular:** Congenital heart disease, patent ductus arteriosus **Other:** Marfanoid habitus, coarse facial features, furrowed forehead, ptosis, thickened eyelids, turtle-backed nails, digital clubbing

*AD* Autosomal dominant, *AR* Autosomal recessive, *SSc* systemic sclerosis

Myhre syndrome is caused by mutations in *SMAD* encoding for SMAD4 protein, a transducer mediating transforming growth factor β (TGF-β) signalling [2–4]. Skin fibroblasts from patients with Myhre syndrome show increased SMAD4 expression, impaired matrix deposition, and altered expression of genes encoding matrix metalloproteinases and related inhibitors. Losartan, an angiotensin-II type 1 receptor blocker but also a (lesser-known) TGF-β antagonist has been shown in vitro to normalize metalloproteinase and related inhibitor transcript levels, and to correct the extracellular matrix (ECM) deposition defect in fibroblasts from these patients [30]. Some patients with aortic pathology associated with Myhre syndrome have already been treated with losartan, with reports of stabilisation of their vasculopathy; but the effect on skin fibrosis has never been described [30–34]. We suggest that further studies could explore losartan (or other therapies acting on the SMAD4 pathway) as a potential targeted therapeutic option for cutaneous fibrosis associated with this rare genetic disease. At the time of writing this report losartan therapy is being considered for the patient described herein.

Several other conditions may also mimic juvenile scleroderma (Table 1). Skin thickening is common to all of these disorders, and may be localized (morphoea-like), or widespread (like diffuse scleroderma) [35–45]. Vasculopathy is frequently observed and should be actively screened for. We highlight for the first time in this case the abnormal nailfold capillaroscopy with similar findings to those observed in SSc. Degenerative cardiac or pulmonary manifestations may also exhibit a secondary inflammatory component, thus posing considerable diagnostic challenges and making it more likely that such patients could be exposed to ineffective but toxic immunosuppression, as illustrated by our case [1, 2, 4, 46–49]. On occasions, autoimmunity has also been described [50–53]. The management and long term outcome of these genetic scleroderma mimics is, however, entirely different and immunosuppression may not be required or may in fact be harmful in some cases [54, 55]. We therefore suggest that genetic testing should be considered in all patients with sclerodermatous skin disease of very young onset (infancy) and recommend screening for vasculopathy (including congenital heart disease and aortopathy) with echocardiography, and non-invasive angiography. Genetic screening for monogenic diseases should also be considered in older patients with scleroderma with atypical clinical course; and in those not responding to conventional immunosuppression.

Regarding the methodology of genetic screening, our case again illustrates the importance of next-generation sequencing (NGS) methodologies in this context. Mainly due to lack of routine NGS methods, initial routine genetic testing of candidate genes by Sanger was performed for this patient. This was a time consuming, costly, and mainly “clinician best guess” driven approach, which resulted in diagnostic delay of several months. Whole exome and genome sequencing and targeted gene panels now allow rapid, simultaneous detection of multiple genes, and are increasingly being used as diagnostic tools and to explore the pathogenesis of monogenic diseases [56–61]. These techniques are particularly useful for screening diseases with overlapping phenotypes. For instance, we (and many others) have used NGS to extensively study monogenic systemic inflammation, with significant diagnostic and therapeutic impact [60, 61]. Similarly, we anticipate that application of NGS genetic screening to cohorts of patients with juvenile scleroderma (in all its forms) may identify a proportion with monogenic disease, and that evidence of tissue inflammation and autoimmunity should not preclude the possibility of a genetic diagnosis for the reasons discussed above.

Understanding the genetic basis of these genetic diseases with sclerodermatous features is not only crucial to secure diagnoses, improve prognostication and to facilitate genetic counselling but may also provide clues to the pathogenesis of sporadic cases. For instance, several of the genetic mimics of scleroderma involve the TGF-β pathway [2, 62–64]. At the cellular level, TGF-β plays potent roles in proliferation, differentiation and apoptosis of many cell types, and therefore unsurprisingly germline mutations in the TGF-β signalling pathway cause various phenotypes affecting the skeletal, muscular, and/or cardiovascular systems [2, 62–65]. TGF-β has also been identified as a regulator of pathological fibrogenesis in juvenile and adult onset systemic sclerosis [64–68]. A wide range of drugs targeting the TGF-β signalling pathways are now available [69–73], and need to be tested for their ability to modulate the phenotypes of both these inherited scleroderma mimics but possibly also for efficacy in addition to anti-inflammatory medication in sporadic systemic sclerosis, given their overlapping pathomechanisms.

## Conclusion

Myhre syndrome is a rare genetic disorder that causes skin thickening and joint contractures, and may be misdiagnosed as juvenile scleroderma (systemic sclerosis). Many other genetic conditions can similarly mimic the clinical manifestations of juvenile scleroderma and should be considered in the differential diagnosis of juvenile scleroderma. Onset in infancy and comorbidities such as structural heart disease, large vessel vasculopathy, dysmorphic features, developmental delay, and hearing loss are important clues to a genetic diagnosis. Clinical application of NGS is likely to transform the genetic diagnostic approach to young patients with scleroderma-like diseases and suggest targeted therapies for some cases. Therapeutic targets for sporadic cases of juvenile scleroderma are also likely to emerge, given the overlapping disease mechanisms for all these conditions leading to vasculopathy, skin and organ fibrosis.

## Supplementary information

**Additional file 1 Supplemental Table** 1. Features of juvenile localised scleroderma, juvenile systemic sclerosis and Myhre syndrome.

## Data Availability

Data sharing is not applicable to this article as no datasets were generated or analysed.

## Abbreviations

ANA: Anti-nuclear antibodies
ECM: Extracellular Matrix
dsDNA: Double-stranded DNA
NGS: Next generation sequencing
SSc: Systemic sclerosis
TGF-β: Transforming growth factor type beta
