# Supplementary material for: A case of Myhre syndrome mimicking juvenile scleroderma
Source: Pediatr Rheumatol Online J. 2020 Sep 11;18:72. doi: 10.1186/s12969-020-00466-1 (PMC7488857; doi:10.1186/s12969-020-00466-1)
Supplement: Supplementary file 1 — Additional file 1 Supplemental Table 1. Features of juvenile localised scleroderma, juvenile systemic sclerosis and Myhre syndrome. [file 12969_2020_466_MOESM1_ESM.docx]

**Supplemental Table 1. Features of juvenile localised scleroderma, juvenile systemic sclerosis and Myhre syndrome.**

|  | **Sclerodermatous skin changes** | **Respiratory involvement** | **Gastrointestinal symptoms** | **Joint symptoms** | **Cardiovascular involvement** | **Dysmorphic features** | **Hearing loss** | **Developmental delay** |
| --- | --- | --- | --- | --- | --- | --- | --- | --- |
| **Juvenile localised scleroderma** | Localised sclerodermatous skin changes | Not commonly observed | Not commonly observed | Arthritis and arthralgia may be observed | Not commonly observed | Not observed | Not observed | Not observed |
| **Juvenile systemic sclerosis** | Diffuse cutaneous skin changes | Interstitial lung disease | Dysphagia,  Reflux,  Oesophageal dysmotility, Gastroparesis,  Gut dysmotility, Strictures | Arthritis  Contractures | Pulmonary arterial hypertension, Cardiomyopathy,  Pericarditis  Myocardial fibrosis  Valvular complications  Arrythmias  Heart failure | Beak-shaped nose  Microstomia | Not observed | Not observed |
| **Myhre syndrome** | Diffuse skin changes | Choanal stenosis, laryngotracheal narrowing, obstructive airway disease, or Restrictive pulmonary disease | Pyloric stenosis, Duodenal strictures, Severe constipation | Contractures | Congenital heart defects, Long- and short-segment stenosis of the aorta and peripheral arteries, Pericardial effusion, Constrictive pericarditis, Restrictive cardiomyopathy, and Arterial hypertension | Short palpebral fissures),  Deeply set eyes,  Short philtrum,  Microstomia,  Prognathism | Common | Common |
